# Reduced scan time and superior image quality with 3D flow MRI compared to 4D flow MRI for hemodynamic evaluation of the Fontan pathway

**DOI:** 10.1038/s41598-021-85936-6

**Published:** 2021-03-22

**Authors:** Friso M. Rijnberg, Hans C. van Assen, Joe F. Juffermans, Lucia J. M. Kroft, Pieter J. van den Boogaard, Patrick J. H. de Koning, Mark G. Hazekamp, Séline F. S. van der Woude, Evangeline G. Warmerdam, Tim Leiner, Heynric B. Grotenhuis, Jelle J. Goeman, Hildo J. Lamb, Arno A. W. Roest, Jos J. M. Westenberg

**Affiliations:** 1 Department of Cardiothoracic Surgery, Leiden University Medical Center, Albinusdreef 2, 2333ZA Leiden, The Netherlands; 2 Department of Radiology, Leiden University Medical Center, Leiden, The Netherlands; 3 Department of Pediatric Cardiology, Leiden University Medical Center, Leiden, The Netherlands; 4 Department of Biostatistics, Leiden University Medical Center, Leiden, The Netherlands; 5 Department of Pediatric Cardiology, Utrecht Medical Center, Utrecht, The Netherlands; 6 Department of Radiology, Utrecht Medical Center, Utrecht, The Netherlands

**Keywords:** Paediatric research, Congenital heart defects

## Abstract

Long scan times prohibit a widespread clinical applicability of 4D flow MRI in Fontan patients. As pulsatility in the Fontan pathway is minimal during the cardiac cycle, acquiring non-ECG gated 3D flow MRI may result in a reduction of scan time while accurately obtaining time-averaged clinical parameters in comparison with 2D and 4D flow MRI. Thirty-two Fontan patients prospectively underwent 2D (reference), 3D and 4D flow MRI of the Fontan pathway. Multiple clinical parameters were assessed from time-averaged flow rates, including the right-to-left pulmonary flow distribution (main endpoint) and systemic-to-pulmonary collateral flow (SPCF). A ten-fold reduction in scan time was achieved [4D flow 15.9 min (SD 2.7 min) and 3D flow 1.6 min (SD 7.8 s), p < 0.001] with a superior signal-to-noise ratio [mean ratio of SNRs 1.7 (0.8), p < 0.001] and vessel sharpness [mean ratio 1.2 (0.4), p = 0.01] with 3D flow. Compared to 2D flow, good–excellent agreement was shown for mean flow rates (ICC 0.82–0.96) and right-to-left pulmonary flow distribution (ICC 0.97). SPCF derived from 3D flow showed good agreement with that from 4D flow (ICC 0.86). 3D flow MRI allows for obtaining time-averaged flow rates and derived clinical parameters in the Fontan pathway with good–excellent agreement with 2D and 4D flow, but with a tenfold reduction in scan time and significantly improved image quality compared to 4D flow.

## Introduction

The Fontan circulation provides a palliative solution for patients with an univentricular heart defect, by directly connecting the caval veins with the pulmonary arteries (PAs), also called the total cavopulmonary connection (TCPC). Today, around 70.000 Fontan patients are alive and this population is expected to double in the next 20 years^1^. Due to the vulnerability of the Fontan physiology to adverse hemodynamics, regular MRI evaluation of the Fontan pathway every 2–3 years is recommended for the early detection of (subclinical) complications^1^. With MRI, important prognostic parameters of both the single ventricle as well as the venous Fontan pathway can be acquired. Parameters of clinical interest of the venous Fontan pathway include the quantification of (1) the pulmonary right-to-left flow distribution^2–4^, associated with exercise capacity^5^, (2) caval flow contribution^6–8^, including the distribution of conduit flow towards the left PA known as hepatic flow distribution (HFD)^9^, and (3) systemic-to-pulmonary collateral flow (SPCF)^2,4,10–12^. Additionally, the inferior vena cava (IVC)-to-extracardiac conduit mismatch (IVC-conduit mismatch) can be quantified to evaluate the adequacy of implanted conduit size^13^.

Although two-dimensional phase-contrast MRI (2D flow MRI) is the current clinical standard for flow quantification^4,12^, measurements at all vessels of interest require extensive, time-consuming planning by a specialized technician^2,10^. ECG-gated 4D flow MRI can measure above mentioned parameters within a single acquisition^2^, by acquiring time-resolved (i.e. usually for 20–30 phases in the cardiac cycle), three-directional velocities within a volume of interest. 4D flow also uniquely allows for visualization of flow patterns within the TCPC^13–15^ and altered, energy-consuming TCPC flow patterns have been associated with adverse outcome^16^. However, clinical applicability of 4D flow remains limited due to long scan times, a significant burden especially for children.

Time-resolved flow acquisition is often required, as flow can be highly pulsatile with significant periodic movement of the region of interest or to obtain flow information at specific phases in the cardiac cycle. In the Fontan pathway, due to the absence of a subpulmonary ventricle, only minimal pulsatility along the cardiac cycle is present^17,18^. Thus, clinical parameters of interest are mostly based on cardiac-cycle averaged flow information.

Consequently, non ECG-gated 3D flow MRI may be sufficient for quantification of cardiac-cycle-averaged parameters. With 3D flow, a single, cardiac-cycle averaged 3D velocity field within a volume of interest is obtained by acquiring data irrespective of the phase in the cardiac cycle, requiring much shorter scan time compared to 4D flow. The feasibility of 3D flow has been theoretically shown for non-pulsatile portal venous flow using time-averaged 4D flow reconstructions^19^. The aim of this study was to compare flow rates in the Fontan pathway and important flow-derived clinical parameters in Fontan patients between 2D, 3D and 4D flow. We hypothesized that 3D flow can provide accurate, equivalent cardiac-cycle averaged parameters compared to 2D and 4D flow, within a significantly shorter scan time which can contribute to a more widespread clinical use.

## Materials and methods

### Study population

The study was approved by the institutional review board of the Leiden University Medical Center. All experiments were performed in accordance with relevant guidelines and regulations. Informed consent was obtained from all subjects and/or their guardians. Thirty-two Fontan patients were prospectively recruited between November 2018 and October 2019 (Table 1). All patients > 8 years without contraindications for MRI were considered eligible for inclusion.

**Table 1:** Patient characteristics.

Male, n (%)	17 (53%)
Height, cm	164.5 (13.4)
Weight, kg	55.5 (14.9)
BSA, m^2^	1.6 (0.3)
Age at MRI, years	17.0 (5.4)
Fontan type (ECC/LT)	29/3
Conduit size (16/18/20 mm)	15/13/1

Values are reported as mean (standard deviation) unless otherwise specified. BSA; body surface area (Haycock), ECC; extracardiac conduit, LT; lateral tunnel, mm; millimeter.

### MRI protocol

**Figure 1:**
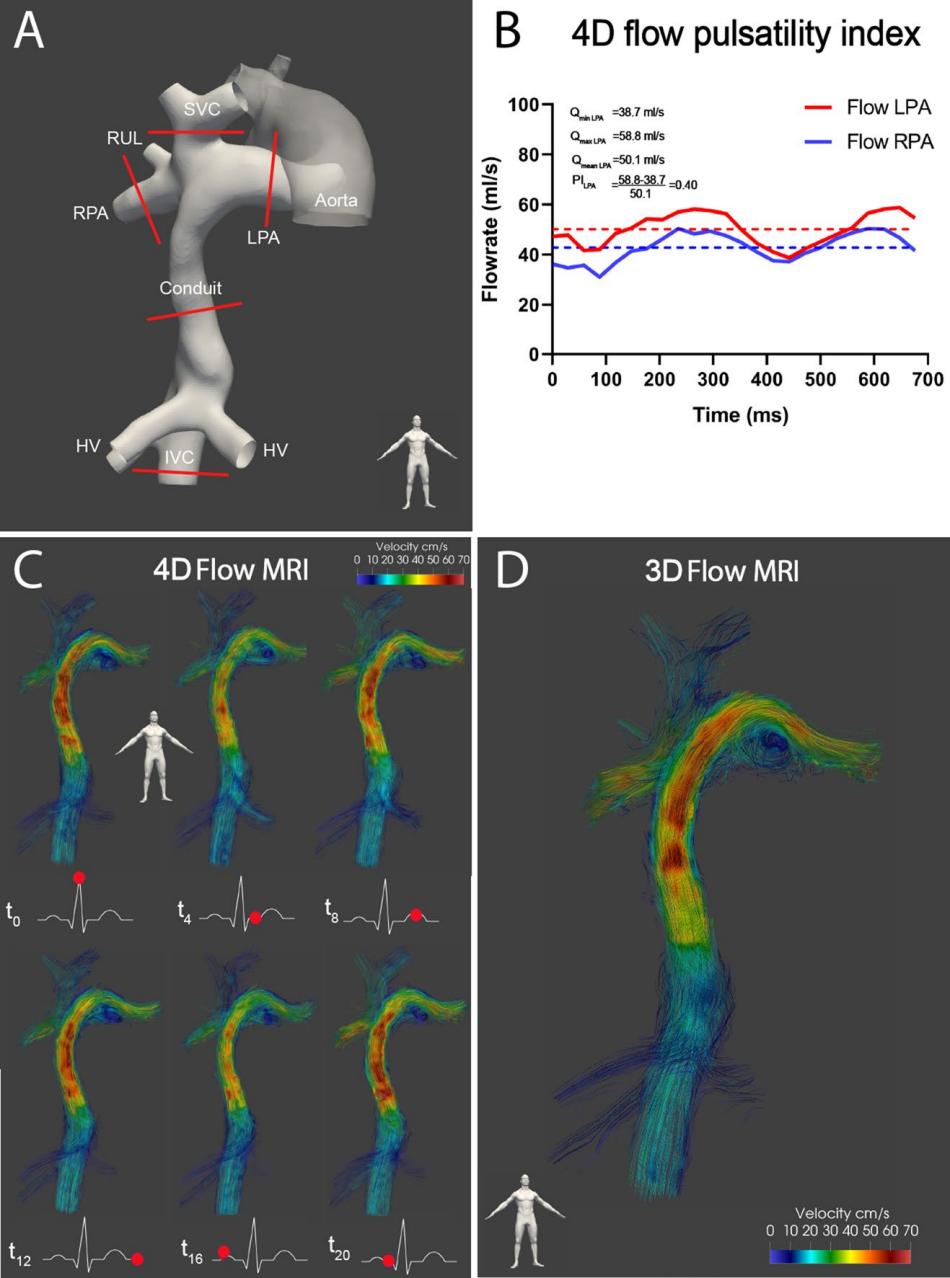
Shows a typical extracardiac conduit Fontan patient (female, 21 years old)**. (A)** The positions of the 2D flow MRI planes are indicated in red. The aorta is shown for orientation purposes only. **(B)** Calculation of the 4D flow pulsatility index for the LPA is shown. **(C)** Streamline visualization is shown for every 4th phase in the cardiac cycle (4D flow) and for the **(D)** single, time-averaged phase acquired with 3D flow. Note how nearly identical flow patterns are captured with 4D and 3D flow, including the appearance of an vortex in the proximal LPA **(C, D)**. RPA/LPA; right/left pulmonary artery, RUL; right upper lobe, IVC/SVC; inferior/superior vena cava, HV; hepatic veins, ms; millisecond, ml; milliliter, MRI; magnetic resonance imaging.

Details of MRI acquisitions are listed in Table 2 (3 T, Ingenia, Philips Healthcare, Best, the Netherlands). Three-directional velocity encoded 2D flow acquisition was acquired at five positions (Fig. 1A): the IVC (below the hepatic veins), Fontan conduit, superior vena cava (SVC) and the left (LPA) and right pulmonary arteries (RPA). For this study, flow was quantified using the through-plane velocity component only as through-plane 2D flow is the current clinical standard. The three-directional velocity encoding sequence in our protocol was chosen to study different research questions not part of this study.

**Table 2:** MRI acquisition details.

	2D flow MRI	3D flow MRI	4D flow MRI
No. of slices (orientation)	1 (per vessel)	27 (coronal)	27 (coronal)
Field of view, mm	350 × 271 × 5	350 × 271 × 56	350 × 271 × 56
ECG-gating	Retrospective	–	Retrospective
No. of reconstructed cardiac phases	24–30	–	24
No. of signal averages	1	1	1
Respiratory compensation	None	Navigator	Navigator
Typical navigator efficiency	–	40–60%	40–60%
Acquired spatial resolution (mm)	1.5 × 1.5 × 5.0	2.4 × 2.4 × 2.4	2.4 × 2.4 × 2.4
Reconstructed spatial resolution (mm)	0.6 × 0.6 × 0.6	2.1 × 2.1 × 2.1	2.1 × 2.1 × 2.1
Acquired temporal resolution, ms (SD)	35.7 (12.0)	–	32.0
Flip angle (°)	10	10	10
TE (ms)	3.5–4.5	4.5	4.5
TR (ms)	5.8–8.0	8.0	8.0
VENC (cm/s)	40–80	80	80
Scan time (minutes)	1.4 (18.6 s)	1.6 (7.8 s)	15.9 (2.7)
Acceleration methods	Segmentation factor 1–2, SENSE factor 1.4, AP direction	SENSE factor 1.5, RL direction, EPI factor 5 Segmentation factor 1	SENSE factor 1.5, RL direction, EPI factor 5, Segmentation factor 1

SD; standard deviation, ms; milliseconds, mm; millimeter, TE; echo time, TR; repetition time, VENC; velocity encoding, SENSE; sensitivity encoding, AP; anterior–posterior, EPI; echo planar imaging readout, ECG; electrocardiogram.

All patients underwent retrospective ECG- and respiratory navigator-gated 4D flow of the Fontan pathway. The acquired volume of interest also covered the right (n = 28) and left (n = 19) pulmonary veins (PVs). Since the 4D flow acquisition was primarily focused on the TCPC, the pulmonary veins were not always present in the field of view. Selecting a larger field of view was not possible in those cases as it would lead to clinically unacceptable 4D flow scan times. Within the same MRI examination, 3D flow was acquired in all patients using an identical acquisition as 4D flow except for the absence of ECG-gating. The 3D flow acquisition was repeated in consecutive order to assess scan-rescan reproducibility (n = 10).

### Image quality and 4D flow pulsatility index

To assess 3D and 4D flow acquisition quality, vessel sharpness and signal-to-noise ratio (SNR) were determined (Supplemental Fig. 1). Relative SNR and vessel sharpness between 3D and 4D flow acquisitions are reported as a ratio. The pulsatility index (Q_max_-Q_min_/Q_mean,_ where Q is flow rate in ml/s) was calculated in the PAs from 4D flow to verify the low-pulsatile character of flow in the Fontan pathway^17,18^.

### Flow quantification and comparisons

Data was pre-processed using automatic background and velocity aliasing correction (CAAS MR Solutions, v5.1, Pie Medical Imaging, Maastricht, the Netherlands). Flow was quantified from 3D and 4D flow using retrospectively placed 2D planes, positioned at corresponding positions of the 2D flow acquisitions (Fig. 1A). Time-averaged flow rates (at the conduit, SVC, RPA and LPA), mean velocity (at the IVC and conduit) and derived parameters were compared between 2D (reference method), 3D and 4D flow acquisitions. PV flow, SPCF and caval flow contribution were compared between 3D and 4D flow only.

### Clinical parameters

Multiple parameters were calculated from the time-averaged flow rate (Q) or mean velocity (v, at IVC and conduit only) measurements: 1) the systemic venous lower-to-upper body flow distribution (Q_conduit_/(Q_conduit_ + Q_SVC_)), 2) the right-to-left pulmonary flow distribution (Q_RPA_/(Q_RPA_ + Q_LPA_)), 3) right, left and total (when both available) SPCF, using the pulmonary estimator (Q_RPV_-Q_RPA_ (n = 28) and Q_LPV_-Q_LPA_ (n = 19))^2,11^. Furthermore, the percentage of 4) IVC-conduit mismatch (excluding lateral tunnel Fontan patients) was quantified by assessing the relative increase in mean velocity from the IVC (below entry of the hepatic veins) towards the extracardiac conduit (v_conduit_-v_IVC_/v_IVC_ *100%)^13^.

### Caval flow contribution

The contribution of SVC and conduit flow towards each PA was quantified from 4D flow using a previously described particle tracing method (n = 28) using open-source Paraview^6,7^. Particles were released from the SVC and conduit and particles arriving at the PAs were recorded^6^. Caval flow contribution was quantified as follows^6,20^:

Conduit-LPA contribution= $\frac{{\mathrm{P}}_{\mathrm{conduit}\_\mathrm{LPA}}}{{\mathrm{P}}_{\mathrm{conduit}\_\mathrm{RPA}}+ {\mathrm{P}}_{\mathrm{conduit}\_\mathrm{LPA}}}$ and SVC-RPA contribution = $\frac{{\mathrm{P}}_{\mathrm{SVC}\_\mathrm{RPA}}}{{\mathrm{P}}_{\mathrm{SVC}\_\mathrm{RPA}}+ {\mathrm{P}}_{\mathrm{SVC}\_\mathrm{LPA}}}$, where P is the number of particles reaching each PA.

The number of missing particles (i.e. the percentage of total emitted particles not reaching one of the PAs) was calculated as a measure of analysis quality^20^. For 3D flow, caval flow contribution was quantified using streamline tracing instead of particle tracing. Under the assumption of (relatively) steady flow, central in this study, streamlines are equal to pathlines.

### Statistical analysis

Fifteen Fontan patients who underwent 2D and 4D flow as part of a previous prospective study^21^ were used for the power calculation, using the right-to-left pulmonary flow distribution as the main endpoint. These patients were not included in this study. The difference between 2D and 3D flow was assumed to be in the same order of magnitude. Based on the mean absolute difference (2.5%) and the standard deviation of this difference (SD, 1.6%) in flow distribution, it was found that a sample size of only 4 patients would provide 90% power (assuming a normal distribution) for detecting equivalence (at equivalence margin 5%) between 2D and 3D flow.

The mean absolute difference (± 1.96 standard error of the mean (SEM)) between methods was the primary endpoint of interest, indicating how much on average *individual* measurements differ (under- or overestimation). In contrast, the mean difference (as assessed by Bland–Altman analysis) reflects how much on average measurements differ on a cohort-level. Since clinicians care for individual patients, the mean absolute difference is considered the more important, informative parameter.

Measurements were considered to be equivalent for the main endpoint right-to-left pulmonary flow distribution when the upper limit (mean absolute difference + 1.96 SEM) was ≤ 5% between methods. Since only evident asymmetric pulmonary flow distribution may require intervention, differences ≤ 5% were considered clinically irrelevant.

Bland–Altman plots and intraclass correlation (ICC) analysis were used to assess agreement between methods and intra- and interobserver variability (moderate < 0.7, good ≥ 0.7–0.9 and excellent > 0.9). Measurements between different acquisition methods were compared using a paired t-test. A p-value < 0.05 was considered statistically significant. Continuous data were presented as mean (standard deviation, SD). Data were analyzed with SPSS 25.0 (IBM-SPSS, Chicago, IL) and Prism 8.0 (Graphpad Software, La Jolla, CA).

## Results

2D flow was incomplete in two patients; in one patient 2D flow of the conduit was of insufficient quality due to excessive breathing artefacts. In the other patient, 2D flow could not be acquired at the end of the scan protocol as the patient did not want to continue the MRI examination. 4D flow was unsuccessful in one 10 year old patient due to excessive movement. 3D flow was successful in all patients. Total scan time of 4D and 3D flow were 15.9 (SD 2.7 min) and 1.6 (SD 7.8 s) minutes, respectively (including a typical navigator efficiency of 50%). Compared with 4D flow, 3D flow showed superior image quality: an improved SNR (mean ratio of SNRs 1.7 (0.8), p < 0.001) and vessel sharpness (mean ratio 1.2 (0.4), p = 0.01). The 4D flow pulsatility index in the RPA and LPA were 0.70 (SD 0.23) and 0.81 (SD 0.34), respectively, corresponding to 14% and 18% of values in healthy persons (Fig. 1B)^22^. Streamline visualization of flow patterns acquired from 4D and 3D flow showed nearly identical flow patterns including the presence of a vortex in the proximal LPA, further illustrating the lack of significant pulsatility in the TCPC (Fig. 1C,D, Supplemental video 3).

### Flow

Results of the comparison of methods are shown in Table 3. Agreement between flow measurements between 2D and 3D flow were good-to-excellent (mean absolute difference 2.3–5.6 ml/s, ICC 0.82–0.96). Mean absolute differences were all similar or smaller compared to mean absolute differences between 2D and 4D flow (mean absolute difference 2.6–6.1 ml/s, ICC 0.78–0.91). 3D flow measured significantly lower conduit and LPA flow in comparison with 2D flow (Table 3). However, mean absolute differences were lower (conduit flow 5.6 vs 6.1 ml/s) or similar (LPA flow 3.3 ml/s) compared to mean absolute differences between 2D and 4D flow, reflecting the larger variation in both under- and overestimation of 4D flow compared with 2D flow.

**Table 3:** Comparison of 2D, 3D and 4D flow MRI measurements in the Fontan pathway.

Flow + Velocity	2D flow MRI(n = 30)	4D flow MRI(n = 31)	3D flow MRI(n = 32)	2D vs 4D			2D vs 3D			4D vs 3D		
				Mean absolute difference	ICC	P value	Mean absolute difference	ICC	P value	Mean absolute difference	ICC	P value
Conduit, ml/s	47.5 (14.3)	48.5 (13.1)	43.2 (11.7)	6.1 (4.3–7.9)	0.81	0.88	5.6 (3.3–7.9)	0.82	**0.01**	6.1 (3.8–8.4)	0.81	**0.002**
SVC, ml/s	22.0 (5.0)	22.2 (5.1)	22.3 (5.3)	2.6 (1.9–3.4)	0.78	0.56	2.3 (1.8–2.8)	0.88	0.39	3.1 (2.3–3.8)	0.74	0.86
RPA, ml/s	34.3 (10.8)	34.3 (10.2)	33.3 (10.1)	3.1 (2.1–4.0)	0.91	0.70	2.6 (1.8–3.3)	0.96	0.55	3.2 (2.3–4.2)	0.91	0.51
LPA, ml/s	27.8 (10.1)	29.0 (11.0)	25.9 (9.8)	3.3 (2.2–4.4)	0.88	0.23	3.3 (2.2–4.4)	0.92	**0.01**	4.2 (2.9–5.5)	0.90	**0.002**
PV right, ml/s (n = 28)	–	45.8 (11.0)	42.8 (11.0)	–	–	–	–	–	–	4.0 (3.0–5.2)	0.93	** < 0.001**
PV left, ml/s (n = 19)	–	35.7 (9.0)	32.4 (8.6)	–	–	–	–	–	–	4.1 (3.0–5.2)	0.87	**0.006**
PV total, ml/s (n = 19)	–	80.9 (17.0)	75.8 (15.1)	–	–	–	–	–	–	7.0 (4.1–9.8)	0.88	**0.02**
IVC, cm/s (n = 28)	15.1 (3.0)	12.9 (2.5)	12.2 (2.6)	2.4 (1.8–3.1)	0.72	** < 0.001**	2.8 (2.2–3.5)	0.80	** < 0.001**	1.5 (1.2–1.8)	0.82	**0.03**
Conduit, cm/s (n = 28)	25.3 (5.7)	23.8 (7.0)	20.9 (6.4)	4.5 (3.6–5.5)	0.78	0.13	4.8 (3.6–6.1)	0.85	** < 0.001**	3.5 (2.4–4.6)	0.83	**0.001**
**Derived parameters**												
SPCF right, ml/s	–	10.5 (7.0)	8.2 (6.8)	–	–	–	–	–	–	3.7 (2.8–4.5)	0.87	**0.002**
SPCF left, ml/s	–	7.2 (5.8)	5.7 (4.1)	–	–	–	–	–	–	3.3 (2.5–4.2)	0.75	0.07
SPCF total, ml/s	–	17.6 (10.7)	14.2 (8.8)	–	–	–	–	–	–	5.0 (3.4–6.7)	0.86	**0.01**
Lower-to-upper body flow distribution, %	67.3 (7.8)	67.9 (7.7)	65.2 (7.4)	3.5 (2.6–4.3)	0.82	0.77	2.9 (2.0–3.8)	0.92	**0.002**	4.1 (3.2–5.0)	0.82	**0.01**
Right-to-left pulmonary flow distribution, %	55.5 (11.4)	54.6 (12.6)	56.5 (11.7)	2.7 (2.1–3.4)	0.96	0.61	2.6 (1.8–3.3)	0.97	** < 0.001**	3.1 (2.2–3.9)	0.97	**0.001**
Conduit-LPA, %	–	51.4 (20.6)	52.9 (26.4)	–	–	–	–	–	**-**	10.0 (7.0–12.9)	0.85	0.52
SVC-RPA, %	–	59.3 (32.3)	65.2 (28.9)	–	–	–	–	–	**–**	13.6 (8.8–18.4)	0.83	0.09
IVC-conduit mismatch, %	75.2 (61.6)	88.1 (60.6)	79.7 (58.5)	28.1 (15.9–40.3)	0.77	0.05	21.6 (15.4–27.8)	0.91	0.18	26.0 (15.9–36.1)	0.81	0.25

Values are reported as mean (± 1.96 standard error of the mean). P-values < 0.05 of the paired t-tests between acquisitions are indicated in bold. ml/s; milliliter per second, IVC/SVC; inferior/superior vena cava, RPA/LPA; right/left pulmonary artery. PV; pulmonary veins, SPCF; systemic-to-pulmonary collateral flow.

When comparing 4D with 3D flow, good-to-excellent agreement was achieved for flow measurements at all vessels (mean absolute differences 3.1–7.0 ml/s, ICC 0.74–0.93, Table 3). 3D flow measured significantly lower flow in the conduit, LPA and PVs, however, mean absolute differences were relatively small and in the same order of magnitude as mean absolute differences between 2D and 3D or 2D and 4D.

### Mean velocity IVC and conduit

Compared with 2D flow, mean velocities were significantly lower in the IVC (3D and 4D flow) and conduit (3D flow, Table 3). Compared with 4D flow, 3D flow measured significantly lower mean velocities in the conduit and IVC.

### Clinical parameters

**Figure 2:**
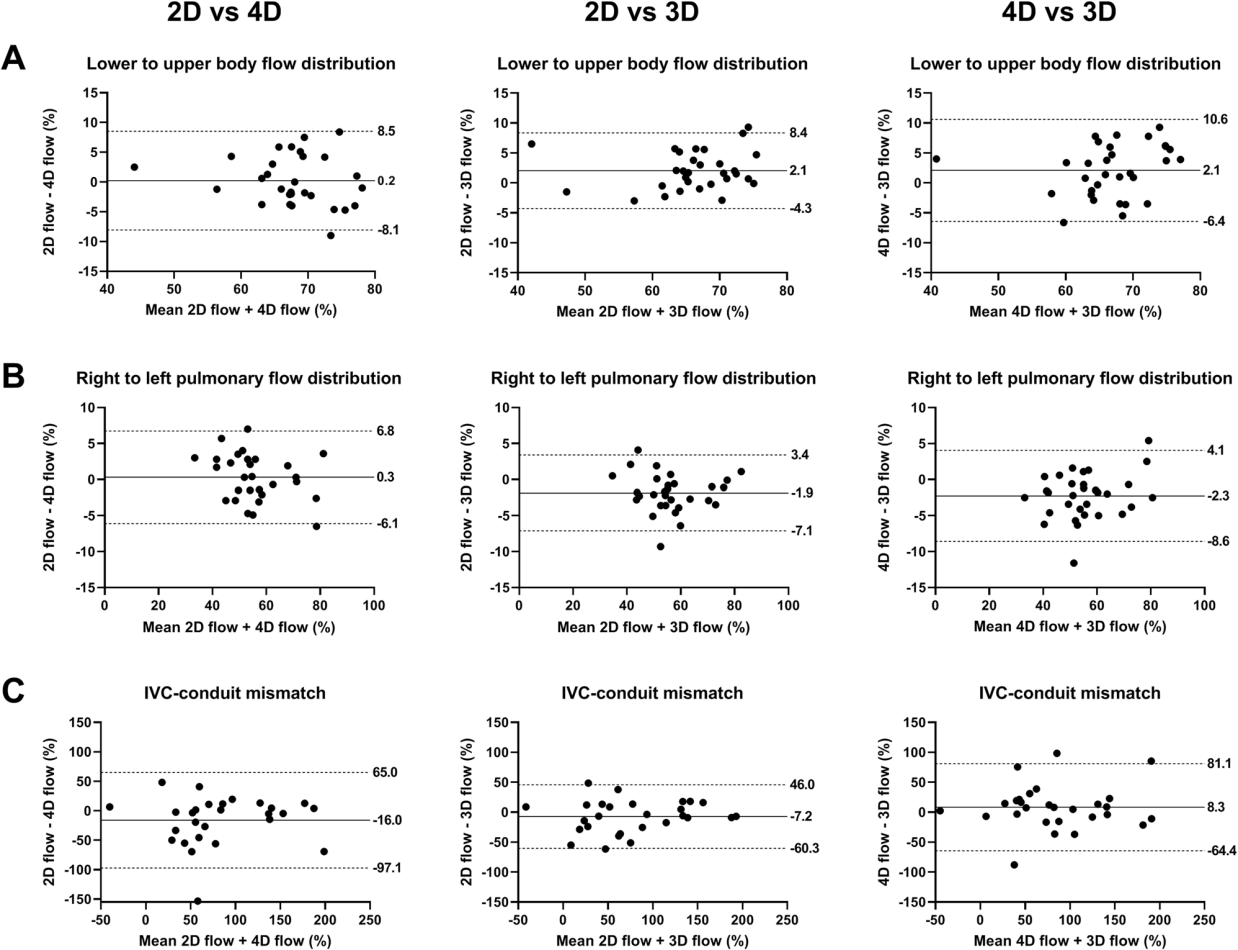
Bland–Altman plots comparing lower-to-upper body flow distribution (**A**), right-to-left pulmonary flow distribution (**B**) and IVC-conduit mismatch (**C**) between 2D, 3D and 4D flow. Mean bias and limits of agreement are shown. IVC; inferior vena cava.

**Figure 3:**
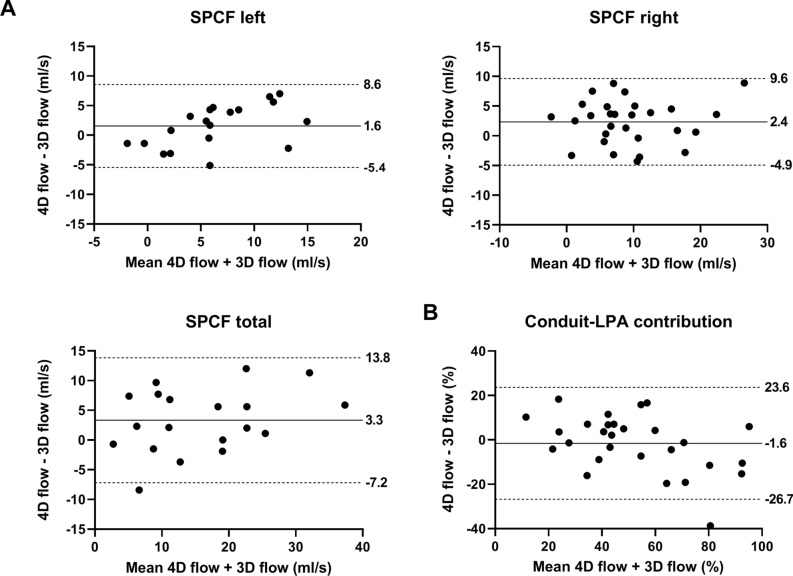
Bland–Altman plots comparing left, right and total SPCF (**A**) and conduit-LPA contribution (**B**) between 4D and 3D flow. Mean bias and limits of agreement are shown. LPA; left pulmonary artery, SPCF; systemic-to-pulmonary collateral flow.

Bland–Altman plots for the comparison between acquisition methods for all clinical parameters are shown in Figs. 2 and 3.

### Pulmonary right-to-left flow distribution and lower-to-upper body flow distribution

Pulmonary right-to-left and lower-to-upper body flow distributions derived from 2D and 3D flow showed significant, but small mean absolute differences (2.6–2.9%) with excellent agreement (ICC 0.92–0.97). Again, mean absolute differences were similar/smaller compared with differences observed between 2D and 4D flow (Table 3).

### Systemic-to-pulmonary collateral flow

SPCF derived from 3D flow and 4D flow showed good agreement (mean absolute differences 3.3–5.0 ml/s, ICC 0.75–0.87), although 3D flow measured significantly lower SPCF in the right lung and total SPCF compared with 4D flow.

### IVC-conduit mismatch

Overall, good-to-excellent agreement was observed between 2D, 3D and 4D flow for IVC-conduit mismatch (ICC 0.77–0.93), with a range of the mean absolute differences of 21.6–28.1%. Of note, when comparing 2D with 3D flow or 2D with 4D flow, differences in IVC-conduit mismatch were particularly highest at low values (IVC-conduit mismatch < 100%), with better agreement at values > 100% (Fig. 2).

### Caval flow contribution

Caval flow contribution derived from 3D flow and 4D flow showed important variability, with mean absolute differences above the equivalence range (upper limit of mean absolute difference 12.9–18.1%). The mean percentage of missing streamlines was significantly higher in 3D flow (conduit 29.8%, SVC 32.2%) compared with missing particles in 4D flow (conduit 18.0%, SVC 24.0%, p < 0.001 and p = 0.01, respectively).

### Intra- and interobserver and scan-rescan analysis

Overall, good-to-excellent intra- and interobserver agreement was shown for all parameters for both 4D (ICC 0.73–0.99) and 3D flow MRI (ICC 0.81–0.99), with mean differences for all flow measurements < 1 ml/s (Supplemental Table 1). Good-to-excellent reproducibility (scan-rescan) of the 3D flow acquisition was achieved for all flow measurements (mean difference 0.0–0.8 ml/s) and derived clinical parameters (mean difference in right-to-left pulmonary flow distribution 0.3%), except for caval flow contribution (ICC 0.73–0.99, Supplemental Table 2).

## Discussion

This study is the first to evaluate the Fontan pathway using non ECG-gated 3D flow MRI and compares measurements with ECG-gated 2D and 4D flow MRI. The main findings are that important cardiac-cycle averaged parameters of clinical interest in Fontan patients can be acquired with 3D flow when compared to gold standard 2D flow MRI, with similar/smaller mean absolute differences for all parameters compared with differences between 2D and 4D flow. A ten-fold reduction in scan time and superior SNR and vessel sharpness was achieved with 3D flow compared to 4D flow. Since clinical important parameters from the venous Fontan pathway are mostly based on cardiac-cycle-averaged flow information, 3D flow acquisition may therefore replace 2D and 4D flow acquisitions to allow for fast, non-invasive hemodynamic flow assessment in the Fontan pathway.

### Clinical relevance of 3D flow for evaluation of the Fontan pathway

Due to the vulnerable state of the Fontan circulation and its dependence on favorable flow hemodynamics, serial surveillance with MRI every 2–3 years is recommended^1^. Phase-contrast MRI flow measurements are an important part of current MRI protocols, and 4D flow MRI is emerging as an alternative to multiple 2D flow measurements in the single ventricle and venous Fontan pathway^2^. However, since the Fontan pathway is characterized by flow with low velocities (usually < 60 cm/s), a dedicated 4D flow for the heart (VENC 150–200 cm/s) and for the Fontan pathway (VENC 60–80 cm/s) is necessary. However, the need for 2 long 4D flow scans prohibit its routine use in standard clinical MRI slots. Furthermore, it provides a significant burden for young children and in general long scan times reduce image quality (e.g. due to patient movement). Importantly, repeating a long 4D flow acquisition in case of problems (e.g. poor quality, patient movement) is often not possible due to time constraints in already long scan protocols. This study shows that 3D flow can be used as an alternative for 4D flow of the Fontan pathway for quantification of multiple, clinically relevant parameters: scan time is reduced from 16 min to only a 1.5 min scan (including a 50% navigator efficiency) with a 100% success rate, thereby easily implementable in current MRI scan protocols. Besides offering an important reduction in scan time, also less post-processing time is required (one delineation per vessel in 3D flow compared to 20–30 (one per cardiac phase) delineations per vessel with subsequent manual adjustments in 4D flow).

### Flow derived parameters

Right-to-left pulmonary flow distribution derived from 3D flow measurements showed excellent agreement with 2D and 4D flow, thereby able to accurately identify patients with (severe) asymmetric flow distributions. This parameter is important in the evaluation of the Fontan pathway, as the combination of flow and morphological information (e.g. PA hypoplasia or stenosis) is essential for clinical decision making regarding possible interventional treatment. An unbalanced right-to-left pulmonary flow distribution has been associated with reduced exercise capacity^23^.

Quantification of systemic-to-pulmonary collateral flow is important in Fontan patients^24^ and can be treated by collateral coiling, making non-invasive assessment of SPCF relevant to identify patients that may require coiling. While 2D and 4D flow MRI can non-invasively quantify SPCF^2,10,11^, both have specific disadvantages. Planning of multiple 2D flow planes is labor intensive and time-consuming to acquire. Furthermore, RPA flow is often acquired distal to the (early branching) right upper lobe PA, leading to errors in calculated pulmonary flow distribution and right SPCF. 4D flow has the advantage of minimal user input (single acquisition), but scan time is long^2^. This study demonstrates that 3D flow overcomes the 2D and 4D flow MRI limitations by allowing for SPCF quantification within a 1.5 min acquisition and, importantly, showed a high reproducibility (scan-rescan). 3D flow measured systematically lower SPCF compared with 4D, but no comparison with gold standard 2D flow derived SPCF could be made in this study. However, a previous study comparing 2D and 4D flow derived SPCF also measured lower SPCF with 2D compared to 4D flow (mean 0.41 vs 0.62 L/min/m^2^, respectively)^2^.

Regular evaluation of extracardiac conduit size adequacy, implanted at 3–5 years of age, is important to ensure an efficient TCPC^25,26^. An early cohort of Fontan patients with 16 mm conduits are reaching adulthood and it is unknown whether these conduits are suitable for adult patients or need replacement for larger conduits^1^. The MRI derived IVC-conduit mismatch percentage (relative increase in mean velocity from IVC towards the conduit) can be useful for identification of patients developing evident IVC-conduit mismatch during somatic growth^13^. Good agreement between 2D and 3D flow was observed, especially for values > 100%. 3D flow therefore allows for the accurate identification of patients with evident mismatch, which can be in the order of > 200%^13^, and may guide when replacement is indicated. Since this parameter is based on mean velocities, some observed variability may be explained by user-variability in correct alignment of the 2D plane perpendicular to the vessel.

Unbalanced caval flow contribution, especially the contribution of conduit flow towards the left PA known as the hepatic flow distribution (HFD)^9^, is associated with pulmonary arteriovenous malformations. While 4D flow MRI has been used for the quantification of caval flow contribution, in our study on average 18–24% of emitted particles did not reach the pulmonary arteries. The percentage of missing particles, related to factors such as velocity noise or low spatial resolution, is often not reported^6,7^, but will influence the accuracy of results^20^. 3D flow showed important variability in measured caval flow contribution (using streamline tracing) compared with 4D flow and thus is unsuitable for this parameter.

Of important note, although some measurements were significantly different between 2D and 3D flow while not between 2D and 4D flow, mean absolute differences were similar/lower for all parameters between 2D and 3D compared to 2D and 4D. This reflects an important observation: there is larger variability in measurements with 4D flow (both under- and overestimation, resulting on average in a non-significant difference) compared to 2D flow, while differences with 3D flow were more systematic (similar/smaller absolute differences, but in general underestimation resulting in a significant difference). Systemic errors can be corrected for which is not possible for random errors, making 3D flow the preferable approach from the perspective of the individual patient.

Of note, 3D and 4D flow acquisitions (end-expiratory navigated acquisitions) are expected to measure lower flowrates in comparison with 2D flow (free-breathing) since flow is lowest during expiration with augmentation during inspiration^27–29^, most pronounced from the lower body. The significant lower mean velocities and/or flow in the IVC and conduit measured with 3D flow compared to 2D flow is therefore in line with this difference in physiology.

## Limitations

The different protocols (free breathing versus respiratory navigator) may have affected flow conditions and subsequent comparisons. Furthermore, 2D-three-directional flow was acquired instead of through-plane 2D flow, which may have introduced differences with clinical standard through-plane 2D flow due to longer acquisition times. Additionally, although respiratory motion suppression improves data quality in 3D and 4D flow, the influence of respiration on flow characteristics in the TCPC are not captured with 2D, 3D or 4D flow measurements.

## Future perspectives

Optimization of 3D flow (e.g. increased spatial resolution, multiple signal averages) can allow for scanning of younger children with smaller vessels. As smaller voxel sizes increase scan time, this optimization is not possible with current 4D flow acquisitions without exceeding clinical acceptable scan times. Importantly, the achieved reduction in scan time from 4D to 3D flow did not require different acceleration techniques (e.g. compressed SENSE), and application of such techniques to the 3D flow sequence can further decrease scan time.

Comparison of 3D- and 4D-flow-derived hemodynamic parameters (e.g. viscous energy loss) are of interest^13,25^, as flow patterns captured with both methods were almost identical.

## Conclusions

3D flow MRI can be used to acquire multiple important cardiac-cycle averaged parameters of interest in the evaluation of the Fontan pathway: right-to-left pulmonary flow distribution, lower-to-upper body flow distribution, SPCF and IVC-conduit mismatch. A ten-fold reduction in scan time with improved image quality was achieved compared to 4D flow MRI. Since primarily cardiac-cycle averaged parameters are of clinical interest, 3D flow may replace time-resolved 2D and 4D flow MRI acquisitions to allow for a fast, accurate hemodynamic evaluation of the Fontan pathway. This could lead to shorter MRI protocols, allow for evaluation of younger Fontan patients, and improve the applicability of state-of-the-art three-dimensional flow imaging in clinical practice.

## Supplementary information

Supplementary figure 1.

Supplementary information.

Supplementary video.

## Data Availability

The data from this study are available from the authors on reasonable request.

## Abbreviations

IVC/SVC: Inferior/superior vena cava
MRI: Magnetic resonance imaging
PA: Pulmonary artery
PV: Pulmonary vein
RPA/LPA: Right/left pulmonary artery
SD: Standard deviation
SNR: Signal-to-noise ratio
SPCF: Systemic-to-pulmonary collateral flow
TCPC: Total cavopulmonary connection
HFD: Hepatic flow distribution
