# Supplementary figures and images for: Reduced scan time and superior image quality with 3D flow MRI compared to 4D flow MRI for hemodynamic evaluation of the Fontan pathway
Source: Sci Rep. 2021 Mar 22;11:6507. doi: 10.1038/s41598-021-85936-6 (PMC7985309; doi:10.1038/s41598-021-85936-6)

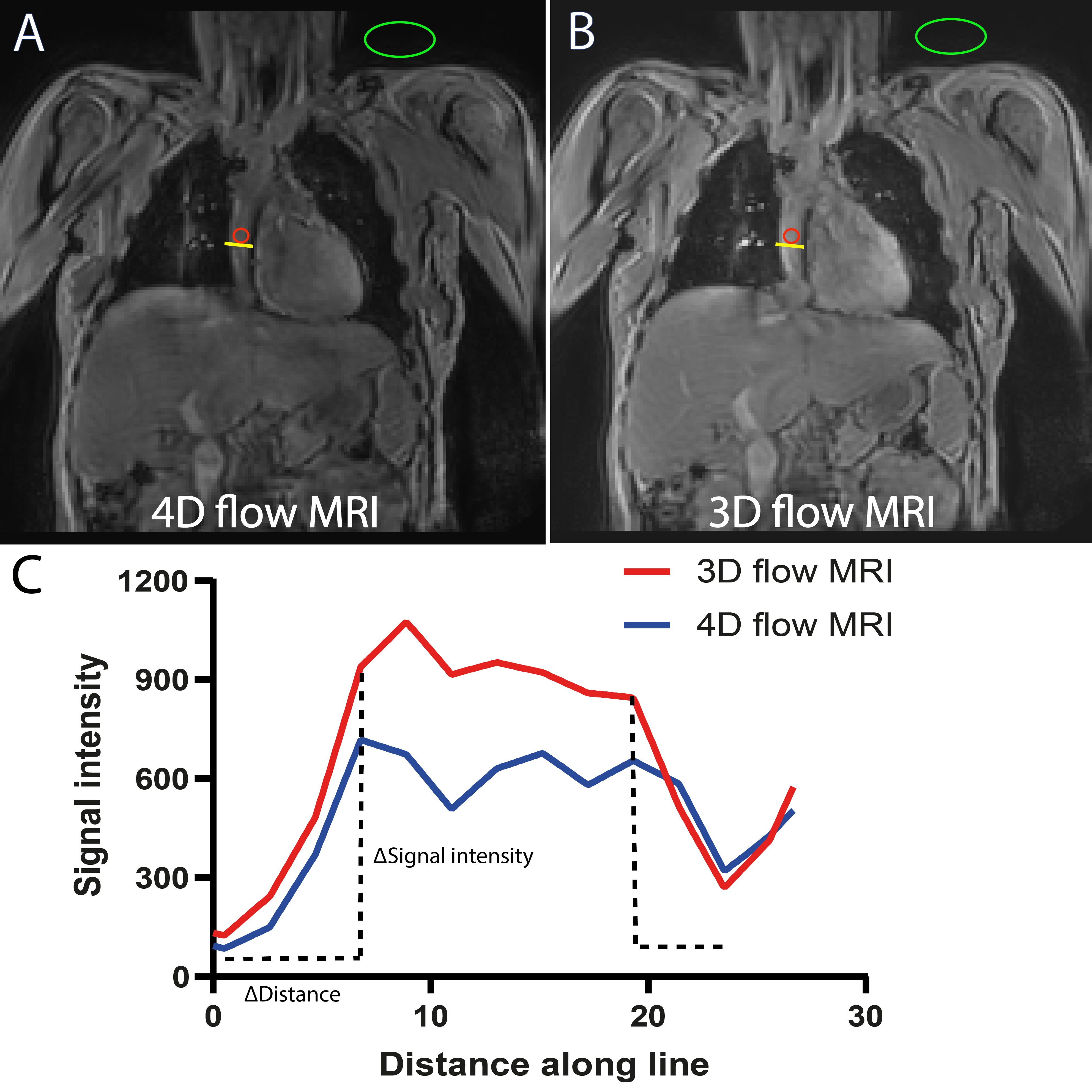

Supplement: Supplementary file 1 — Supplementary figure 1. [file 41598_2021_85936_MOESM1_ESM.tif]
