# Supplementary material for: Reduced scan time and superior image quality with 3D flow MRI compared to 4D flow MRI for hemodynamic evaluation of the Fontan pathway
Source: Sci Rep. 2021 Mar 22;11:6507. doi: 10.1038/s41598-021-85936-6 (PMC7985309; doi:10.1038/s41598-021-85936-6)
Supplement: Supplementary file 2 — Supplementary information. [file 41598_2021_85936_MOESM2_ESM.pdf]

Reduced scan time and superior image quality with 3D flow MRI compared to 4D flow MRI for hemodynamic evaluation of the Fontan pathway

Friso M. Rijnberg[a], MD, Hans C. van Assen[b], PhD, Joe F. Juffermans[b], Msc, Lucia J. Kroft[b], MD, PhD, Pieter J. van den Boogaard[b], BSc, Patrick J.H. de Koning[b], MSc, Mark G. Hazekamp[a], MD, PhD, Séline F.S. van der Woude[c], MD, Evangeline G. Warmerdam[e], MD, Tim Leiner[f], MD, PhD, Heynric B. Grotenhuis[e], MD, PhD, Jelle J. Goeman[d], PhD, Hildo J. Lamb[b], MD, PhD, Arno A.W. Roest[c], MD, PhD, Jos J.M. Westenberg[b], PhD

[a]Department of Cardiothoracic surgery, [b]Department of Radiology, [c]Department of Pediatric cardiology, [d]Department of Biostatistics, Leiden University Medical Center, Leiden, the Netherlands

[e]Department of Pediatric cardiology, [f]Department of Radiology, Utrecht Medical Center, Utrecht, the Netherlands

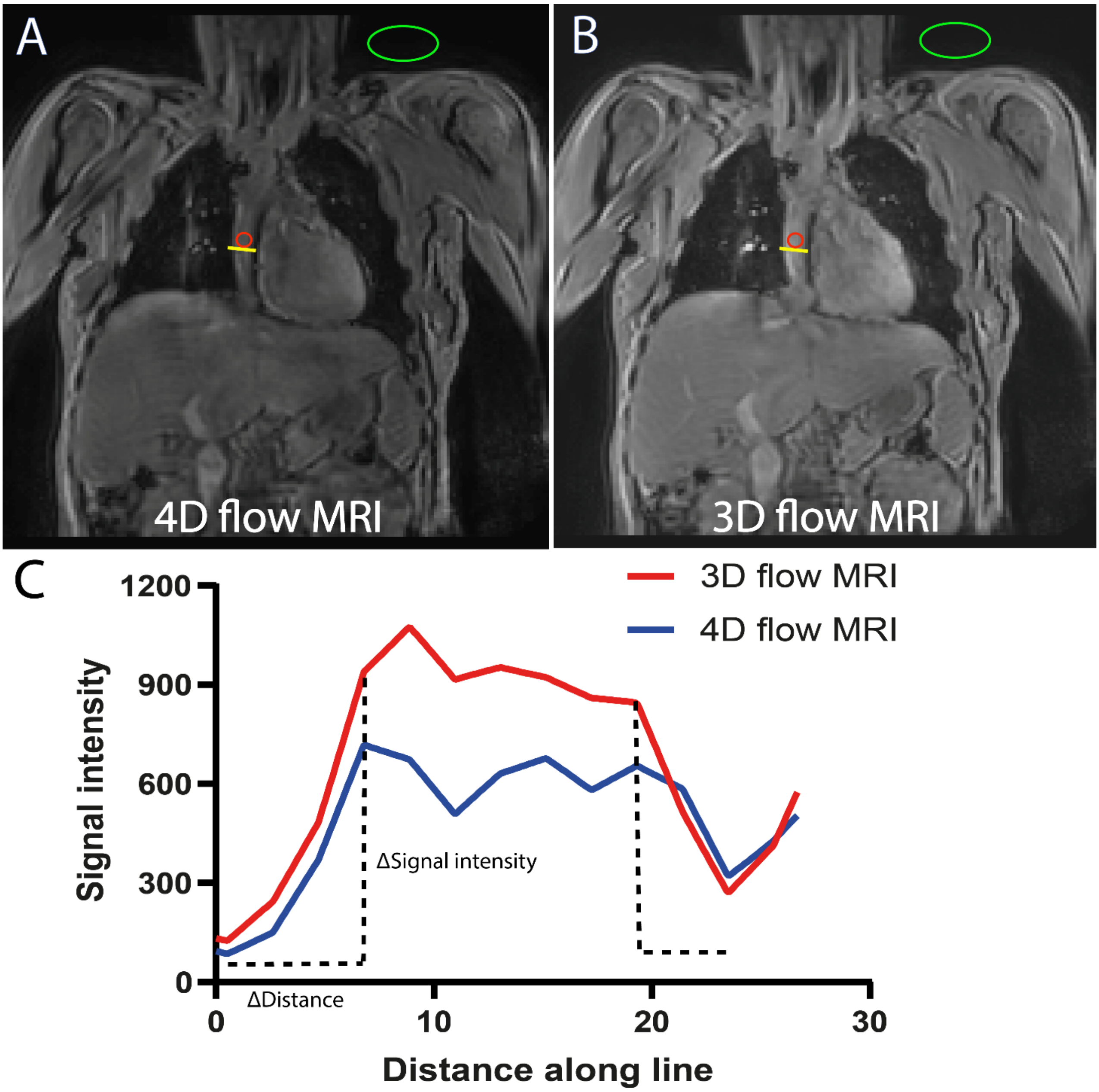

Legend Supplemental Figure 1: vessel sharpness and SNR.

Magnitude images of **(A)** 4D (phase 0) and **(B)** 3D flow MRI acquisitions are shown for a Fontan patient with an extracardiac conduit.

(B) The vessel sharpness was quantified by placing a measurement line perpendicular to the Fontan conduit. **(C)** A line intensity profile was determined and the slope of this profile was calculated at either side of the vessel and then averaged, resulting in an overall vessel sharpness measurement. The slopes were calculated by dividing the difference in minimal and maximal signal intensity by the difference in distance. **(A-B)** SNR was determined by placement of standardized region of interests (ROIs) in the Fontan conduit (red, ± 0.5cm^2) and in the air (green, ± 5cm^2). The SNR was calculated by dividing the mean signal intensity in the ROI in the Fontan conduit by the standard deviation of the signal intensity in the ROI in the air. The same positions of lines and ROIs were used in both datasets per patient.

Legend Supplemental Video 1

Video 1 shows the time-resolved pathline representation of 4D flow MRI derived blood flow in the TCPC of a 21-year old female extracardiac conduit Fontan patient (left). Only minimal pulsatility is present, appreciated by the only gradual changes in velocities within the conduit and SVC. The major flow patterns in the TCPC, including the presence of a vortex in the proximal LPA, remain relatively stable along the cardiac cycle. Streamline visualization of blood flow in the TCPC from 3D flow (right) are also shown. Note how nearly identical flow patterns are captured using these method.

Supplemental Table 1. Inter- and intraobserver analysis of 3D and 4D flow MRI measurements (n=10)

	**4D flow MRI**	**4D flow MRI intra**	**4D flow MRI inter**	**3D flow MRI**	**3D flow MRI intra**	**3D flow MRI inter**	**Paired t-test** P-value			
**Flow + Velocity**							**4D vs $4D_{intra}$**	**4D vs $4D_{inter}$**	**3D vs $3D_{intra}$**	**3D vs $3D_{inter}$**
Conduit, ml/s	47.5 (10.8)	47.8 (10.6)	45.8 (9.6)	46.0 (10.7)	46.8 (11.1)	45.4 (9.8)	0.72	0.06	0.11	0.36
SVC, ml/s	23.9 (6.9)	24.1 (6.2)	24.0 (6.4)	23.9 (7.3)	23.4 (6.5)	23.6 (7.9)	0.80	0.98	0.23	0.72
RPA, ml/s	35.9 (11.0)	36.4 (11.6)	35.0 (10.9)	34.5 (10.4)	34.8 (10.6)	34.9 (10.9)	0.56	**0.02**	0.10	0.23
LPA, ml/s	28.2 (13.2)	27.9 (12.8)	29.1 (14.9)	26.6 (12.5)	26.9 (12.1)	27.6 (13.7)	0.74	0.22	0.64	0.36
PV right, ml/s	47.9 (9.7)	47.9 (8.7)	47.0 (11.1)	44.4 (10.8)	44.5 (11.5)	44.2 (11.0)	0.94	0.40	0.89	0.56
PV left, ml/s	36.8 (10.9)	36.1 (8.9)	37.1 (12.1)	33.1 (10.8)	33.3 (9.8)	32.6 (10.7)	0.50	0.75	0.76	0.42
PV total, ml/s	83.5 (19.0)	84.0 (14.4)	84.0 (19.6)	77.8 (18.3)	77.7 (18.1)	76.8 (18.5)	0.67	0.66	0.75	0.36
IVC, cm/s	13.1 (2.4)	13.3 (2.4)	13.7 (2.3)	12.5 (2.5)	12.6 (2.5)	13.3 (2.5)	0.32	**0.03**	0.71	0.07
Conduit, cm/s	23.1 (7.4)	25.0 (7.1)	26.0 (7.2)	22.6 (6.6)	23.5 (8.0)	23.3 (7.4)	0.15	**0.02**	0.37	0.42
**Derived parameters**										
SPCF right, ml/s	12.0 (10.1)	11.5 (11.6)	11.9 (10.9)	9.9 (8.1)	9.6 (8.2)	9.2 (8.5)	0.61	0.97	0.42	**0.03**
SPCF left, ml/s	8.7 (6.3)	8.2 (7.8)	8.0 (7.0)	6.5 (4.0)	6.4 (3.8)	5.1 (5.4)	0.61	0.21	0.88	0.15
SPCF total, ml/s	20.7 (13.1)	19.7 (13.8)	20.0 (14.7)	16.4 (9.5)	16.0 (8.9)	14.5 (9.7)	0.47	0.67	0.51	0.07
Lower-to-upper body flow distribution, %	66.5 (6.9)	66.3 (6.5)	65.7 (6.3)	65.9 (4.9)	66.6 (4.9)	65.9 (6.7)	0.80	0.07	0.22	0.97
Right-to-left pulmonary flow distribution, %	56.9 (15.3)	57.2 (16.3)	56.0 (16.3)	57.1 (13.8)	57.0 (13.3)	56.6 (14.2)	0.71	0.11	0.74	0.67
IVC-Conduit mismatch, %	76.5 (52.5)	88.6 (44.3)	89.7 (42.6)	84.2 (53.5)	87.8 (52.3)	75.9 (49.8)	0.31	0.22	0.72	0.42

Values are reported as mean (SD). ml/s; milliliter per second, MRI; magnetic resonance imaging, IVC/SVC; inferior/superior vena cava, RPA/LPA; right/left pulmonary artery. Right-to-left pulmonary flow distribution: RPA flow/ LPA flow *100%

PV; pulmonary veins, SPCF; systemic-to-pulmonary collateral flow (pulmonary vein – pulmonary artery flow).

Supplemental Table 1. Continued intraobserver analysis

	4D vs $4D_{intra}$			3D vs $3D_{intra}$		
	Bland-Altman		Intraclass correlation	Bland-Altman		Intraclass correlation
**Flow + Velocity**	Mean difference	LoA	ICC	Mean difference	LoA	ICC
Conduit, ml/s	- 0.3	-4.6 – 4.0	0.98	-0.7	-3.3 – 1.8	0.99
SVC, ml/s	- 0.2	-3.7 -3.4	0.97	0.6	-2.2 - 3.3	0.98
RPA, ml/s	-0.4	-4.6 – 3.8	0.98	-0.4	-1.6 – 0.9	0.99
LPA, ml/s	0.2	-4.0 – 4.4	0.99	-0.2	-3.3 – 2.8	0.99
PV right, ml/s	0.1	-4.5 – 4.6	0.97	-0.1	-2.1 – 2.0	0.99
PV left, ml/s	0.7	-5.6 – 7.0	0.95	-0.2	-3.3 – 3.0	0.99
PV total, ml/s	0.8	-9.1 – 10.7	0.96	-0.2	-3.7 – 3.3	0.99
IVC, cm/s	-0.2	-1.6 – 1.1	0.96	-0.1	-1.9 – 1.7	0.94
Conduit, cm/s	-1.9	-9.3 – 5.5	0.85	-0.8	-6.4 – 4.7	0.93
**Derived parameters**						
SPCF right, ml/s	0.5	-5.0 – 6.0	0.97	0.3	-2.0 – 2.6	0.99
SPCF left, ml/s	0.5	-5.2 – 6.2	0.92	0.1	-3.1 – 3.2	0.92
SPCF total, ml/s	1.0	-6.9 – 8.8	0.96	0.4	-3.2 – 4.0	0.98
Lower-to-upper body flow distribution, %	0.1	-4.1 – 4.4	0.95	-0.8	-4.1 – 2.6	0.93
Right-to-left pulmonary flow distribution, %	-0.3	-4.5 – 3.9	0.99	0.1	-1.9 – 2.0	0.99
IVC-conduit mismatch, %	-12.1	-81.9 – 57.7	0.73	-3.9	-63.4 – 55.6	0.85

Values are reported as mean (SD). ml/s; milliliter per second, MRI; magnetic resonance imaging, LoA; limits of agreement, IVC/SVC; inferior/superior vena cava, RPA/LPA; right/left pulmonary artery. Right-to-left pulmonary flow distribution: RPA flow/ LPA flow *100%
PV; pulmonary veins, SPCF; systemic-to-pulmonary collateral flow (pulmonary vein – pulmonary artery flow).

Supplemental Table 1. Continued interobserver analysis

	**4D vs 4D$_{inter}$**			**3D vs 3D$_{inter}$**		
	Bland-Altman		Intraclass correlation	Bland-Altman		Intraclass correlation
**Flow + Velocity**	Mean difference	LoA	ICC	Mean difference	LoA	ICC
Conduit, ml/s	1.8	-3.2 – 6.7	0.96	0.7	-3.6 – 5.0	0.97
SVC, ml/s	0.0	-2.7 – 2.6	0.98	0.3	-4.5 – 5.0	0.95
RPA, ml/s	0.9	-1.1 – 2.9	0.99	-0.5	-2.7 – 1.7	0.99
LPA, ml/s	0.9	-5.1 – 3.3	0.99	-0.9	-6.8 – 5.0	0.97
PV right, ml/s	1.0	-5.7 – 7.6	0.95	0.2	-2.0 – 2.4	0.99
PV left, ml/s	0.2	-4.7 – 4.2	0.98	0.5	-2.9 – 3.9	0.99
PV total, ml/s	0.7	-9.0 – 10.4	0.97	1.0	-3.5 – 5.5	0.99
IVC, cm/s	-0.6	-2.0 – 0.8	0.93	-0.8	-3.3 – 1.7	0.84
Conduit, cm/s	-2.8	-9.0 – 3.4	0.85	-0.6	-5.2 – 4.0	0.95
**Derived parameters**						
SPCF right, ml/s	0.1	-7.3 – 7.4	0.94	0.7	-0.9 – 2.3	0.99
SPCF left, ml/s	0.7	-2.3 – 3.7	0.97	1.4	-4.1 – 6.8	0.81
SPCF total, ml/s	0.7	-9.4 – 10.8	0.94	2.1	-3.5 – 7.6	0.94
Lower-to-upper body flow distribution, %	0.8	-1.7 – 3.4	0.98	0.0	-6.0 – 5.9	0.82
Right-to-left pulmonary flow distribution, %	0.9	-2.2 – 4.0	0.99	0.5	-6.6 – 7.6	0.97
IVC-conduit mismatch, %	-13.2	-75.1 – 48.8	0.77	8.3	-52.8 – 69.4	0.82

Values are reported as mean (SD). ml/s; milliliter per second, MRI; magnetic resonance imaging, LoA; limits of agreement, IVC/SVC; inferior/superior vena cava, RPA/LPA; right/left pulmonary artery. Right-to-left pulmonary flow distribution: RPA flow/ LPA flow *100%, PV; pulmonary veins, SPCF; systemic-to-pulmonary collateral flow (pulmonary vein – pulmonary artery flow).

Supplemental table 2. Scan-rescan analysis of 3D flow MRI.

	Measurements		3D vs 3Drescan flow				
	**3D flow MRI (N=10)**	**3D flow MRI rescan (n=10)**	T-test	Bland-Altman		Intraclass correlation	Mean absolute difference
			P-value	Mean difference	LoA	ICC	(± 1.96 SEM)
**Flow + Velocity**							
Conduit, ml/s	46.9 (9.6)	46.2 (10.2)	0.50	0.6	-4.7 – 6.0	0.96	2.2 (1.2-3.2)
SVC, ml/s	23.5 (4.8)	23.5 (4.3)	0.97	0.0	-4.7 – 4.7	0.86	1.7 (0.6-2.7)
RPA, ml/s	36.5 (8.4)	36.4 (8.6)	0.86	0.1	-3.7 – 3.9	0.97	1.6 (1.1-2.2)
LPA, ml/s	29.5 (9.9)	29.4 (8.5)	0.92	0.1	-4.5 – 4.6	0.97	1.9 (1.2-2.6)
PV right, ml/s	44.9 (11.8)	44.7 (11.3)	0.88	0.1	-4.9 – 5.1	0.98	1.6 (0.4-2.8)
PV left, ml/s (n=6)	35.3 (10.3)	35.1 (9.8)	0.85	0.2	-4.3 – 4.6	0.98	1.6 (0.5-2.8)
PV total, ml/s (n=6)	84.4 (16.4)	83.6 (17.6)	0.22	0.8	-2.0 – 3.6	0.99	1.3 (0.5-2.0)
IVC, cm/s	13.2 (2.4)	13.4 (2.2)	0.39	-0.3	-2.6 – 1.9	0.89	0.9 (0.4-1.4)
Conduit, cm/s	20.9 (5.3)	21.2 (5.5)	0.66	-0.3	-3.7 – 3.2	0.95	1.4 (0.7-2.0)
**Derived parameters**							
SPCF right, ml/s	8.3 (7.5)	8.3 (8.5)	0.99	0.0	-5.1 – 5.2	0.95	2.0 (1.0-3.0)
SPCF left, ml/s (n=6)	5.6 (2.7)	5.9 (2.9)	0.71	-0.3	-4.4 – 3.7	0.73	1.5 (0.5-2.5)
SPCF total, ml/s (n=6)	16.6 (6.3)	17.6 (8.0)	0.37	-1.0	-5.8 – 3.8	0.94	1.9 (0.6-3.2)
Lower-to-upper body flow distribution, %	66.4 (3.8)	66.1 (4.1)	0.72	0.3	-4.9 – 5.6	0.77	2.1 (1.0-3.1)
Right-to-left pulmonary flow distribution, %	55.8 (8.1)	55.5 (8.2)	0.62	0.3	-3.7 – 4.4	0.97	1.6 (0.8-2.4)
Conduit-LPA, %	57.4 (28.5)	48.6 (18.4)	**0.04**	8.8	-14.3 – 31.9	0.83	11.7 (6.3-17.0)
SVC-RPA, %	69.3 (20.2)	73.5 (29.3)	0.53	-4.2	-44.0 – 35.7	0.67	15.6 (7.7-23.5)
IVC-conduit mismatch, % (n=9)	66.5 (63.2)	60.10 (45.9)	0.42	6.4	-38.5 – 51.3	0.91	17.3 (7.3-27.3)

* Values are in ml/s unless otherwise specified. Values are reported as mean (SD). LoA; limits of agreement, IVC/SVC; inferior/superior vena cava, RPA/LPA; right/left pulmonary artery. PV; pulmonary veins, SPCF; systemic-to-pulmonary collateral flow (pulmonary venous – pulmonary artery flow). SEM; standard error of the mean
